# Onychophagia in Obsessive–Compulsive Disorder (OCD): Prevalence and Clinical Characterisation

**DOI:** 10.3390/brainsci15111228

**Published:** 2025-11-15

**Authors:** Luca Pellegrini, Gabriele Di Salvo, Gianluca Rosso, Giuseppe Maina, Umberto Albert

**Affiliations:** 1 Department of Medicine, Surgery and Health Sciences, UCO Clinica Psichiatrica, University of Trieste, 34127 Trieste, Italy; ualbert@units.it; 2 Department of Mental Health, Psychiatric Clinic, Azienda Sanitaria Universitaria Giuliano-Isontina—ASUGI, 34149 Trieste, Italy; 3 School of Life and Medical Sciences, University of Hertfordshire, Hatfield AL10 9AB, UK; 4 Centre for Psychedelic Research and Neuropsychopharmacology, Imperial College London, London NW1 5QH, UK; 5 Department of Neurosciences “Rita Levi Montalcini”, University of Turin, 10126 Turin, Italy; gabriele.disalvo@unito.it (G.D.S.); gianluca.rosso@unito.it (G.R.); giuseppe.maina@unito.it (G.M.); 6 Psychiatric Unit, San Luigi Gonzaga University Hospital, Orbassano, 10043 Turin, Italy

**Keywords:** OCD, Obsessive-Compulsive, onychophagia, ASD, autism

## Abstract

**Introduction:** Onychophagia, commonly known as nail-biting, is a chronic and repetitive behaviour disorder characterised by a compulsive/habitual nature. Obsessive–compulsive disorder (OCD) and onychophagia present a noteworthy intersection in clinical psychiatry. With a paucity of clinical investigations on this topic, we decided to perform a study on onychophagia in OCD. **Materials and Methods:** In this cross-sectional investigation, the sample comprised patients (aged 18 years and older) having a primary diagnosis of OCD (DSM-5) and a score on the Yale–Brown Obsessive–Compulsive Scale of at least 16 (moderate OCD). Individuals were referred to the Department of Neuroscience at the University of Turin. Analysis of the data was performed using JASP (Version 0.16.3), a freely available statistical programme created by the University of Amsterdam (JASP Team, 2022). Statistical value was set at *p* < 0.05. **Results:** Our sample consisted of 603 individuals with OCD, and onychophagia was present in 52 of the cases, with a prevalence of 8.6% (95% CI: 6.5–11.2%). Individuals with OCD and onychophagia had some specific clinical features compared to patients with only OCD. The main difference was detected in terms of the presence of autism spectrum disorder (ASD): in the group of patients having OCD and onychophagia, a prevalence of ASD as high as 96.2% was identified, compared to 18.0% in the OCD-without-onychophagia group. **Discussion:** Onychophagia is a relatively common problem in patients with OCD, with almost one individual out of ten experiencing this issue. OCD and onychophagia, when both present, might define a peculiar clinical phenotype with specific characteristics. The extremely high frequency of ASD in patients with OCD and onychophagia (96.2%) might be very useful information for clinicians, who should pay particular attention to screening for autism in this cohort of individuals.

## 1. Introduction

### Onychophagia

Onychophagia, commonly known as nail-biting, is a chronic and repetitive behaviour disorder characterised by the compulsive biting of one’s fingernails or toenails [1,2]. It is a prevalent condition, affecting an estimated 20–30% of the population across all age groups with different degrees of severity [3,4]. The onset of nail-biting typically occurs during childhood and adolescence, with the fingernails being the most-commonly affected area [1,5]. Although frequent in childhood, onychophagia can persist into adulthood only in a minority of cases (1–5%), with some individuals developing it as a habit [6]; this suggests the existence of specific and as-yet poorly understood risk factors.

Onychophagia is categorised in the Diagnostic and Statistical Manual of Mental Disorders, 5th edition [7], inside the “Other Specified Obsessive-Compulsive and Related Disorders” section. The DSM-5 additionally categorises onychophagia as a recurring body-focused repetitive behaviour disorder, including lip biting and cheek chewing. The category of body-focused repetitive behaviour disorders also includes conditions such as trichotillomania (hair pulling) and excoriation (skin-picking) [2,8]. These disorders are characterised by recurrent, irresistible urges to engage in self-grooming behaviours that result in physical damage [2,8]. The diagnostic criteria for onychophagia are fulfilled when patients exhibit clinically significant distress or impairment in social and occupational functioning, which cannot be more accurately attributed to trichotillomania, excoriation disorder, stereotypic movement disorder, or non-suicidal self-injury. These individuals must have made several unsuccessful efforts to inhibit their nail-biting [2].

Clinically, onychophagia is marked by an inability to resist the urge to bite one’s nails, often preceded by rising tension or emotional discomfort and followed by relief or gratification. It is frequently triggered or exacerbated by stress, anxiety, boredom, or perfectionistic tendencies [9]. The clinical presentation of onychophagia can vary, with some individuals exhibiting a more severe and persistent form of the behaviour, while others may engage in nail-biting intermittently [8,10]. Chronic and excessive nail-biting can lead to various complications, including damage to the cuticle, permanent nail deformities, bleeding around the nail margin, and secondary bacterial infections [11,12]. This maladaptive behaviour can cause a variety of medical complications, including paronychia (inflammation of the nail fold), gingival abscesses, and increased oral bacterial colonization [1]. In severe cases, it may also result in osteomyelitis (infection of the bone) and other serious infections [13,14,15]. The compulsive nature of nail-biting can also negatively impact an individual’s quality of life, leading to social and functional impairments [3,4,16].

Overall, onychophagia is a complex and multifaceted condition that requires a comprehensive approach to assessment and management. Increased awareness, early intervention, and a combination of behavioural and pharmacological therapies can be effective in addressing this common yet often-overlooked disorder [2,3,17,18]. Precise diagnosis requires thorough history-taking and physical examination, since patients seldom arrive with nail-biting or nail picking as their primary concern. The effective care of onychophagia requires both non-pharmacological and pharmaceutical interventions, requiring a multidisciplinary approach that includes dermatologists, internists, paediatricians, psychiatrists, and dentists; it often involves an approach combining behavioural interventions, such as habit reversal therapy and cognitive–behavioural therapy, with pharmacological treatments when necessary [2,17,18]. Pharmacological therapies, including selective serotonin reuptake inhibitors (SSRIs) and tricyclic antidepressants, have been used to address the disorder [5,19]. Research has also explored the efficacy of N-acetylcysteine (NAC) as a potential treatment option for onychophagia and other BFRBs [2,20,21].

Although onychophagia is classified in “Other Specified Obsessive-Compulsive and Related Disorders” and it is a body-focused repetitive behaviour (BFRB), its overlap with OCD remains insufficiently clarified.

Obsessive–compulsive disorder and onychophagia, in fact, present an interesting intersection in clinical psychiatry, highlighting the complexity of compulsive behaviours and the neurobiological mechanisms that underlie them. OCD is marked by obsessive thoughts and compulsive actions, leading to significant disruption in daily functioning across social, occupational, or other important areas due to these symptoms [22,23]. A particular subset of these compulsions includes body-focused repetitive behaviours (BFRBs) like onychophagia, which necessitates further examination regarding their prevalence, clinical implications, and treatment methodologies.

Although isolated studies exist on onychophagia as a stand-alone habit or within the spectrum of BFRBs, its characterisation in patients with OCD remains underexplored, which justifies the relevance of the present study. Patients exhibiting onychophagia have been found to score higher on measures of obsessive–compulsiveness, particularly those who perceive their nail-biting as a significant problem [5]. Onychophagia was the second-most common disorder found in a study investigating the comorbidity between OCD and impulse-control disorders (ICDs) [24], with a current rate of 10%, right after skin-picking disorder (current rate of 12.8%). In another study, the authors showed that having OCD determined an increased risk of more than four times of having nail-biting or pathological skin-picking [25]. On the other end, controversy exists between reports on the two conditions, with the investigation by Pacan and colleagues (2014) [26] finding no correlation between onychophagia and OCD: among the participants who bit their nails during their lifetime, five persons (3.1%) met criteria for OCD, while in the group without onychophagia, OCD occurred in nine persons (5.0%).

Considering the lack of studies on onychophagia in OCD, and given the high prevalence of both conditions, there is an unmet need to further elucidate the clinical and psychopathological components of this comorbidity. In this study, we aim to identify the prevalence of onychophagia in OCD (expected to be significant and around 10%) and, for the first time, explore possible sociodemographic and clinical characteristics associated with this particular repetitive behaviour in OCD.

## 2. Materials and Methods

### 2.1. Data

The data of this current cross-sectional investigation were obtained from a larger longitudinal observational research study including individuals with OCD [27,28]. The sample comprised consecutive outpatients (aged 18 years and older) having a primary diagnosis of OCD (DSM-5) and a score on the Yale–Brown Obsessive–Compulsive Scale of at least 16 (moderate OCD). Individuals were referred to the Department of Neuroscience at the University of Turin. Subjects with a previous or present diagnosis of schizophrenia, psychotic disorders, or organic brain syndrome were excluded from the study. Other comorbidities were included in the study, provided that OCD was the principal diagnosis. Data were collected in in-person visits by using a semi-structured interview as per previous studies from our research group [27]. The information collected in the systematic face-to-face interview included the following areas:

- Sociodemographic information, such as age, gender, marital status, educational attainment, and employment position.
- Clinical features such as the severity of obsessive–compulsive disorder, as measured by the Yale–Brown Obsessive–Compulsive Scale symptom checklist.
- Psychiatric comorbidities assessed during a clinical interview by using DSM-5 (or DSM-IV equivalent) criteria and by using the Italian version of the Structured Clinical Interview for DSM-5 Axis I Disorders. Particular attention was devoted to affective disorders, obsessive–compulsive disorders, and similar related disorders (e.g., body-focused repetitive behaviours (BFRBs).

The semi-structured interviews were carried out by psychiatrists with experience with OCD. Onychophagia and other BFRBs were assessed by clinical observation, inquiry, and confirmation according to DSM-5 (or equivalent) criteria. Different BFRBs were identified according to the specific clinical features. ASD was evaluated according to DSM-5 diagnostic (or equivalent) criteria during the structured clinical interview (SCID-5); subthreshold autistic traits without functional impairment were not coded as ASD.

### 2.2. Psychometric Instruments

All clinical measures were administered in their Italian versions. The Structured Clinical Interview for DSM-5 Disorders [29] was used to establish DSM-5 diagnoses through a semi-structured clinical interview conducted by experienced psychiatrists. The severity of obsessive–compulsive symptoms was rated with the Yale–Brown Obsessive–Compulsive Scale (Y-BOCS) [30,31]. The Y-BOCS contains 10 clinician-rated items (0–4 each; total 0–40) and shows high internal consistency (Cronbach’s α = 0.88–0.91) and excellent inter-rater reliability (ICC > 0.85) across languages, including the Italian version (α = 0.91). Depression and anxiety symptoms were assessed using the Hamilton Depression Rating Scale [32] and the Hamilton Anxiety Rating Scale [33].

### 2.3. Ethics

All participants who engaged with our inpatient and outpatient treatments submitted written informed permission, which was approved by our ethics committee, permitting the use of their clinical data for research purposes under the condition of anonymous handling. A formal request was submitted to our ethics committee (Comitato Etico Interaziendale Azienda Ospedaliera-Universitaria San Luigi Gonzaga di Orbassano) for access to the clinical records of all patients with OCD who granted consent; the protocol received approval from the institutional ethics committee (protocol number 0007375).

### 2.4. Statistical Analysis

Analysis of the data was performed using JASP (Version 0.16.3), a freely available statistical programme created by the University of Amsterdam (JASP Team, 2022). Assumption checks were performed to assess normality using the Shapiro–Wilk test. Paired-samples Student t-tests were used to examine variations where normality could be assumed. In cases where departures from normality were observed, the Mann–Whitney U test was adopted. Chi-squared test statistics were employed to detect differences on categorical variables. Statistical value was set at *p* < 0.05. We used the Benjamini–Hochberg false discovery rate (FDR) correction, applying a liberal exploratory threshold (q < 0.10) to balance Type I and Type II errors and to retain potentially meaningful clinical signals. To address multiple univariate comparisons, *p*-values were corrected for false discovery rate (FDR) using the Benjamini–Hochberg procedure, with a liberal exploratory threshold of q < 0.10 due to the correlated nature of the clinical variables examined.

## 3. Results

Our sample consisted of 603 individuals with OCD, and onychophagia was present in 52 of the cases during their lifetime, with a prevalence of 8.6% (95% CI: 6.5–11.2%). No differences were found in terms of sociodemographic factors between the groups with and without onychophagia (see Table 1).

In terms of clinical characteristics, no differences were found with respect to the severity of OCD symptoms (Y-BOCS), depressive symptoms (HAM-D), and anxiety symptoms (HAM-A) (see Table 2). The rates of comorbidity with major depressive disorder and anxiety disorders were comparable among the two groups, and the duration of untreated illness (DUI) was also similar, as well as a the age at disorder onset.

Interestingly, OCD symptoms manifested earlier in patients with onychophagia compared to patients without onychophagia (15.2 years versus 16.9 years, *p* = 0.04). Moreover, an abrupt onset was more frequent in the onychophagia group than in the group without this specific behaviour (38.5% versus 25.8% of the samples, *p* = 0.04). Among all the Y-BOCS types of obsessions and compulsions, statistically significant differences between the two groups were present with regards to symmetry obsessions and repetition compulsions, with both of these specific symptoms being more common in the individuals experiencing onychophagia (see Table 2). A significant statistical difference was detected in terms of the presence of autism spectrum disorder (ASD): in the group of patients having OCD and onychophagia, a prevalence of ASD of 96.2% was identified, compared to 18.0% in the OCD without onychophagia group (see Table 2). After FDR correction, the associations between onychophagia and both autism spectrum disorder (ASD; q = 0.016) and symmetry obsessions (q = 0.080) remained statistically significant. The link with repetition compulsions (q = 0.107) approached significance, suggesting a similar trend. Other comparisons did not reach the adjusted threshold (all q > 0.12).

## 4. Discussion

Onychophagia is a relatively common problem in patients with OCD, with almost one individual out of ten experiencing this issue. The prevalence of onychophagia in OCD found in this study is very similar to the one obtained by Grant and colleagues (10%) [24]. On the other end, the null association between OCD and onychophagia reported by Pacan et al. (2014) [26] likely reflects important methodological and sampling differences compared with our study. First, Pacan and colleagues examined a community sample using a lifetime history of nail-biting (any frequency), whereas we studied a clinical OCD cohort, in which pathological onychophagia (impairing/repetitive, body-focused behaviour) is more likely to be expressed and detected. Second, their assessment was based primarily on self-reports, while our diagnoses were clinician-established (e.g., distinguishing habitual nail-biting from BFRB that meets impairment criteria). Taken together, these differences plausibly explain the discrepancy: onychophagia may not co-vary with OCD at the population level, but a clinically meaningful BFRB subtype emerges within specialist OCD samples.

Intriguingly, we located a very high frequency of ASD in the individuals with OCD and onychophagia (96.2%), and this, although a preliminary finding, might reflect a stereotypical component of the nail-biting behaviour. This piece of information may be very useful for clinicians, who should pay particular attention to screening patients for autistic spectrum disorder when OCD and onychophagia are both present. We could even hypothesise that onychophagia might be a clinical marker or proxy for ASD in OCD.

Moreover, individuals with onychophagia were characterised by symptoms such as repetition compulsions and symmetry obsessions that could be more typical of ASD than pure OCD itself. The higher frequency of repetition compulsions in the OCD + onychophagia group is in line with the nature of pathological nail-biting, which might acquire an habitual element over time. The fact that symmetry obsessions were more common in subjects with onychophagia and OCD could suggest a greater tendency toward perfectionism and the need for control and routine. These converging findings suggest a broader “symmetry–orderliness” phenotype within the OCD–onychophagia subgroup, which might partly overlap with autistic-spectrum rigidity or stereotypy.

Individuals with onychophagia showed, in an unadjusted analysis, a reduced age at symptoms onset and a more prominent and abrupt type of disorder onset compared to individual without onychophagia. This could be, hypothetically, in relation to the problems that arise from pathological nail-biting, which could make the symptoms more evident and easy to recognise. Moreover, the consequent health and damage issues of this body-focused behaviour could prompt earlier medical assistance and help-seeking, although it should be noted that no significant differences were detected in terms of the duration of untreated illness between the OCD-only group and the OCD + onychophagia group.

Therefore, it is important to bear in mind the peculiar clinical phenotype of OCD when it is in comorbidity with onychophagia.

The relationship between OCD and onychophagia could extend beyond mere symptom overlap, prompting consideration of translational and neurobiological factors. Research indicates that the neuroanatomical underpinnings of OCD involve dysfunction within cortico-striato-thalamic circuits, which are similarly implicated in the manifestation of BFRBs, including nail-biting [34,35]. This neurocognitive perspective suggests that both conditions may stem from similar pathophysiological processes characterised by heightened anxiety and inadequate impulse control. Such neurobiological overlap informs an understanding that treatments effective for OCD, particularly selective serotonin reuptake inhibitors (SSRIs), may also be beneficial for those with onychophagia [19]. Recognising onychophagia within the context and conceptual framework of OCRDs could inform treatment protocols, indicating the importance of a multifaceted approach, including cognitive–behavioural therapy (CBT) tailored to address both the obsessions/compulsions associated with OCD and the habitual nature of onychophagia [19]. Studies showed the efficacy of behavioural therapies in reducing symptoms of onychophagia, providing evidence for positive outcomes achieved through integrative treatment modalities consisting of CBT enhanced with habit-reversal training [4]. Furthermore, psychiatric interventions that include medication and have been shown to reduce symptoms of OCD can also improve the habitual component of onychophagia [19]. Ultimately, addressing these interconnected issues within a unified framework could enhance therapeutic efficacy and improve patient outcomes among individuals suffering from these conditions.

### Limitations

This study has several limitations that should be acknowledged. First, its cross-sectional design prevents causal inferences regarding the relationship between OCD and onychophagia; longitudinal studies are needed to clarify directionality. Second, all participants were recruited from a single clinical centre, which may over-represent more severe or complex cases (including those with neurodevelopmental comorbidity), thus reducing external validity. Third, although diagnostic assessments were clinician-based and structured (SCID-5), the study did not employ additional standardised tools specifically validated for body-focused repetitive behaviours. Fourth, we did not use an a priori power calculation nor regression analyses to account for possible confounders. Fifth, we relied on clinical diagnosis (although made by psychiatrists with experience with OCD and related disorders) without using reliability measures (e.g., kappa rate between interviewers). Despite these limitations, the large sample size and use of structured diagnostic interviews strengthen confidence in the robustness of the main findings. Nevertheless, the present study contributes novel evidence on the clinical characterisation of OCD patients with onychophagia, highlighting a distinct phenotype marked by prominent symmetry and stereotypy features, and it provides a foundation for future longitudinal and multicentre investigations to confirm and extend these findings.

## 5. Conclusions

Onychophagia is a fairly common body-focused behaviour in OCD with some specific clinical features.

Future studies employing a longitudinal design should be conducted to further elucidate and underpin the relationship between OCD and onychophagia. Treatment trials could be of particular use for exploring possible therapeutic options for patients with this comorbidity.

In conclusion, our findings suggest that onychophagia in OCD should not be regarded as a mere habit, but rather as a clinical marker of potential value for diagnosis and treatment, particularly in the early recognition of ASD.

## Figures and Tables

**Table 1:** Sociodemographic characteristics.

Variable	OCD + Onychophagia N = 52	OCD Without Onychophagia N = 551	Total Sample N = 603	*p*-Value (Between-Group Differences) *
*Females—N (%)*	23 (44.2 )	252 (45.7)	275 (45.6)	*p* = 0.84
*Employed—N (%)*	24 (46.2 )	267 (48.5)	291 (48.3)	*p* = 0.75
*Family history of OCD—N (%)*	11 (21.2)	109 (19.8)	120 (19.9)	*p* = 0.81
*Age—mean years (SD)*	32.8 (12.3)	35.3 (12.5)	35.0 (12.5)	*p* = 0.21
*Education—mean years (SD)*	13.0 (3.9)	12.7 (3.5)	12.7 (3.5)	*p* = 0.60

* Comparative analyses of the sociodemographic variables between the groups (OCD + Onychophagia vs. OCD without Onychophagia). Chi-squared test statistics were employed to detect differences in categorical variables. Paired-samples Student t-tests were used to examine variations in continuous variables where normality could be assumed; in cases where departures from normality were observed, the Mann–Whitney U test was adopted. Statistical value was set at *p* < 0.05.

**Table 2:** Selected clinical characteristics.

Variable	OCD + Onychophagia N = 52	OCD Without Onychophagia N = 551	Statistic	*p*-Value (Between-Group Differences) *	Effect Size
*Y-BOCS—mean score (SD)*	24.3 (6.9)	24.3 (6.2)	Mann–Whitney U test	*p* = 0.99	d (95% CI) = 0.00 (−0.18–0.18)
*HAM-D—mean score (SD)*	11.1 (6.6)	11.0 (6.4)	Mann–Whitney U test	*p* = 0.97	d (95% CI) = 0.02 (−0.16–0.19)
*HAM-A—mean score (SD)*	11.5 (6.7)	11.9 (6.6)	Mann–Whitney U test	*p* = 0.77	d (95% CI) = −0.06 (−0.24–0.12)
*Age at OC symptoms onset—mean years (SD)*	15.2 (8.7)	16.9 (8.1)	Mann–Whitney U test	***p*** **= 0.03**	d (95% CI) = −0.21 (−0.40–−0.02)
*Age at disorder (OCD) onset—mean years (SD)*	20.7 (9.3)	22.0 (8.5)	Mann–Whitney U test	*p* = 0.11	d (95% CI) = −0.14 (−0.32–0.04)
*Duration of untreated illness (DUI)—mean months (SD)*	129.6 (154.0)	109.2 (116.0)	Mann–Whitney U test	*p* = 0.99	d (95% CI) = 0.15 (−0.05–0.34)
*Type of onset*			Chi-squared test	***p*** **= 0.04**	ln (OR) (95% CI) = 0.59 (0.00–1.18) (abrupt)
*Abrupt—N (%)*	20 (38.5)	142 (25.8)			
*Insidious—N (%)*	32 (61.5)	409 (74.2)			
*Comorbidity with MDD*			Chi-squared test	*p* = 0.83	ln (OR) (95% CI) = 0.08 (−0.61–0.77)
*Yes—N (%)*	12 (23.1)	120 (21.8)			
*No—N (%)*	40 (76.9)	431 (78.2)			
*Comorbidity with Anxiety Disorders*			Chi-squared test	*p* = 0.36	ln (OR) (95% CI) = 0.32 (−0.43–1.07)
*Yes—N (%)*	12 923.1)	99 (18.0)			
*No—N (%)*	40 (76.9)	452 (82.0)			
*Comorbidity with ASD*			Chi-squared test	***p*** **< 0.001**	ln (OR) (95% CI) = 4.74 (3.31–6.17)
*Yes—N (%)*	50 (96.2)	99 (18.0)			
*No—N (%)*	2 (3.8)	452 (82.0)			
*Symmetry obsessions*			Chi-squared test	***p*** **= 0.01**	ln (OR) (95% CI) = 0.75 (0.14–1.36)
*Yes—N (%)*	18 (34.6)	110 (20.0)			
*No—N (%)*	34 (65.4)	441 (80.0)			
*Repetition compulsions*			Chi-squared test	***p*** **= 0.02**	ln (OR) (95% CI) = 0.69 (0.10–1.27)
*Yes—N (%)*	34 (65.4)	268 (48.6)			
*No—N (%)*	18 (34.6)	283 (51.4)			

Y-BOCS: Yale–Brown Obsessive–Compulsive Scale; HAM-D: Hamilton Rating Scale for Depression; HAM-A: Hamilton Anxiety Rating Scale; OC: obsessive–compulsive; MDD: major depressive disorder; and ASD: autism spectrum disorder. d: Cohen’s d and ln (OR): log odds ratio. * Comparative analyses of the clinical variables between the groups. Assumption checks were performed to assess normality using the Shapiro–Wilk test. In cases where departures from normality were observed, the Mann–Whitney U test was adopted. Chi-squared test statistics were employed to detect differences in categorical variables. Statistical value was set at *p* < 0.05.

## Data Availability

The data presented in this study are available on request from the corresponding author (due to privacy concerns).
